# A PAD-Based Unmanned Aerial Vehichle Route Planning Scheme for Remote Sensing in Huge Regions

**DOI:** 10.3390/s23249897

**Published:** 2023-12-18

**Authors:** Tianyi Shao, Yuxiang Li, Weixin Gao, Jiayuan Lin, Feng Lin

**Affiliations:** 1 College of Computer Science, Sichuan University, Chengdu 610065, China; shaotianyi30@stu.scu.edu.cn (T.S.); liyuxiang1@stu.scu.edu.cn (Y.L.); gaoweixin1@stu.scu.edu.cn (W.G.); 2 School of Geographical Sciences, Southwest University, Chongqing 400715, China; joeylin@swu.edu.cn

**Keywords:** remote sensing, PAD, path planning, UAV

## Abstract

Unmanned aerial vehicles (UAVs) have been employed extensively for remote-sensing missions. However, due to their energy limitations, UAVs have a restricted flight operating time and spatial coverage, which makes remote sensing over huge regions that are out of UAV flight endurance and range challenging. PAD is an autonomous wireless charging station that might significantly increase the flying time of UAVs by recharging them in the air. In this work, we introduce PADs to simplify UAV-based remote sensing over a huge region, and then we explore the UAV route planning problem once PADs have been predeployed throughout a huge remote sensing region. A route planning scheme, named PAD-based remote sensing (PBRS), is proposed to solve the problem. The PBRS scheme first plans the UAV’s round-trip routes based on the location of the PADs and divides the whole target region into multiple PAD-based subregions. Between adjacent subregions, the UAV flight subroute is planned by determining piggyback points to minimize the total time for remote sensing. We demonstrate the effectiveness of the proposed scheme by conducting several sets of simulation experiments based on the digital orthophoto model of Hutou Village in Beibei District, Chongqing, China. The results show that the PBRS scheme can achieve excellent performance in three metrics of remote sensing duration, the number of trips to charging stations, and the data-storage rate in UAV remote-sensing missions over huge regions with predeployed PADs through effective planning of UAVs.

## 1. Introduction

The progress of automation control and data processing technology has significantly contributed to the rapid growth of unmanned aerial vehicle (UAVs) remote-sensing technology [1,2,3]. This technology offers numerous advantages, including the ability to operate without the constraints of complex ground conditions, improved mobility, cost efficiency, increased safety, and impressive performance in spatial scale and data accuracy. UAVs are generally categorized into two main types: fixed-wing UAVs and rotary-wing UAVs. Rotary-wing UAVs have distinct benefits over fixed-wing UAVs, with them notably their capability for vertical takeoff and landing, as well as hovering [4]. In comparison, fixed-wing UAVs require more specific launch conditions. The unique features of vertical takeoff, landing, and hovering make rotary-wing UAVs the preferred option in situations where these capabilities are essential, particularly in remote-sensing missions.

**Figure 1:**
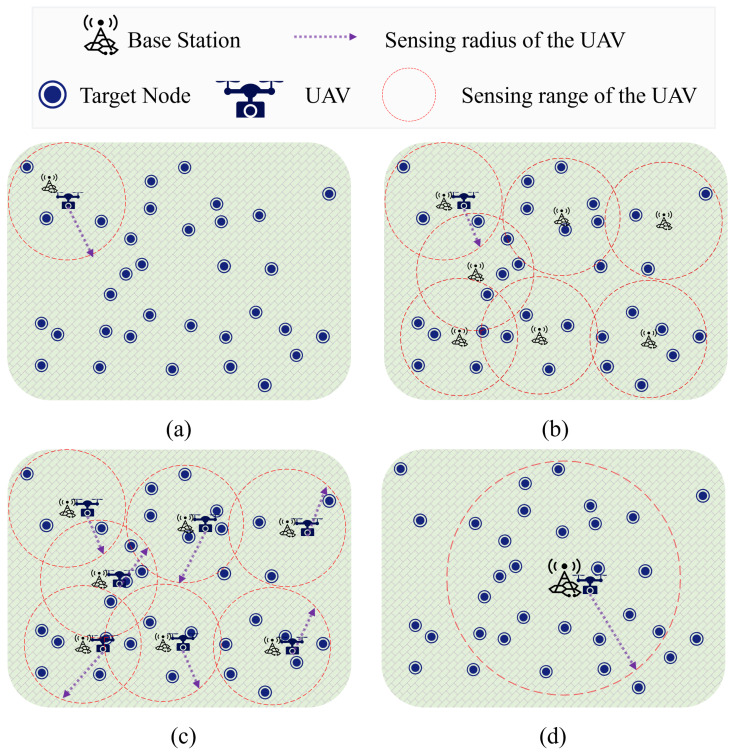
The drawbacks of flying a single UAV over a huge region and the three main ways to solve them. Where (**a**) depicts the initial remote sensing setup with a single UAV and a lone base station, (**b**) depicts the multi-flight approach with a single UAV, (**c**) illustrates multi-UAV cooperation for executing remote sensing missions and (**d**) shows a single UAV with increased battery capacity for executing remote sensing missions.

The problem of UAV route planning has become a hotspot of research in numerous related domains due to the energy limitation in terms of flight endurance and range of UAVs, thereby presenting substantial research potential [5]. The problem of UAV route planning holds significant relevance in the field of remote-sensing engineering, as it directly impacts the quality and efficiency of remote sensing. However, because of the limitations of battery capacity and its own weight, a single UAV’s flight-operating time and spatial coverage cannot match the needs of huge-region remote-sensing missions. As shown in Figure 1a, the UAV cannot travel to all target points in a single flight. Currently, there are three main methods for a huge-region remote-sensing mission:

(1) Single-UAV with multiple flights: As shown in Figure 1b, this method means a single UAV covers the entire region with multiple flights [6].
(2) Multi-UAV cooperation: This method employs multiple UAVs to simultaneously cover the entire region [7]. Figure 1c shows a paradigm of this method.
(3) Single-UAV with increased battery capacity: As shown in Figure 1d, this method enhances the UAV’s battery capacity, thereby enabling it to survey the entire region in a single flight [8].

However, all of these methods have drawbacks that make it difficult to achieve highly effective remote-sensing observations. In the first method, the UAV has to make several trips back and forth between the target region and the base station (BS) for energy replenishment. To make matters worse, the BS must be relocated, as shown in Figure 1b, to cover more target points, thereby leading to a prolonged duration between the collections of images. The second method operates by simultaneously introducing multiple UAVs, thereby significantly reducing the mission execution time compared to the first method. However, as shown in Figure 1c, the cost of executing a remote-sensing mission is significantly higher when multiple UAVs are used at once, in addition to the setup of multiple BSs. Additionally, the concurrent operation of multiple UAVs raises the risk of collisions. Regarding the third method, theoretically, enhancing the UAV’s battery capacity could improve its endurance and broaden its remote-sensing range. However, a larger battery increases the UAV’s weight and volume, thereby leading to a higher energy-consumption rate during flight.

With the advancement of wireless power transfer technologies, researchers have developed a novel wireless charging station, known as PAD, for charging UAVs [9,10]. Consequently, an energy-depleted UAV can instead head to a nearby PAD for energy replenishment. This approach enhances the UAV’s operational time and spatial coverage, as well as reduces the frequency of repetitive flights to the BS for charging. PAD technology has demonstrated its effectiveness in wireless rechargeable sensor networks (WRSNs). In these networks, PADs can be strategically placed in advance to recharge UAVs, which then act as mobile chargers for the sensor nodes.

**Figure 2:**
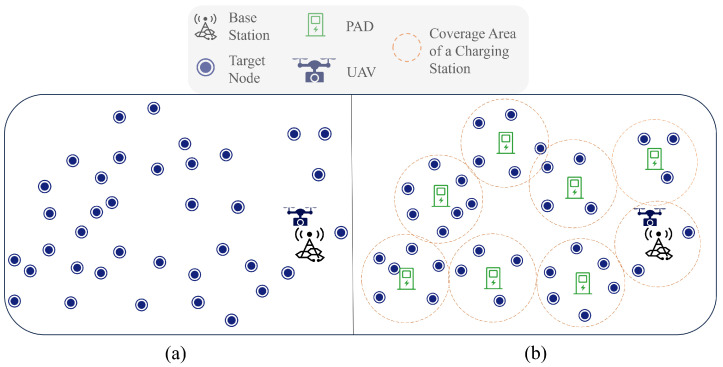
(**a**) represents the initial remote-sensing region. (**b**) represents the remote-sensing region after the introduction of PAD.

Inspired by the PAD-based WRSNs where the UAV charges nodes, we introduce PADs in the field of remote sensing and study the PAD-based UAV remote-sensing route-planning problem for huge regions in this work. As shown in Figure 2, by deploying PADs in the target observation region, a single UAV can accomplish remote-sensing observation missions over a huge monitoring region in a single flight. Both UAVs in WRSNs and UAVs in remote-sensing missions need to depart from a base station, perform a mission, and return. To simplify the problem, we analogize the target points in remote-sensing missions to sensor nodes. In our scheme, an energy-depleted UAV can visit a nearby PAD for replenishment. Therefore, current UAV remote-sensing route-planning methods are not suitable for our mission. Similarly, the existing mobile-charger scheduling algorithms in WRSNs, designed to prioritize the most energy-depleted nodes, do not align with our requirements. Moreover, in our case, it is crucial for the UAV to consider its limited storage capacity and the energy used for turning during remote-sensing missions. In conclusion, while the introduction of PADs brings a new method to accomplish UAV remote-sensing missions over huge regions, it also brings new challenges for PAD-based UAV remote-sensing route planning.

In this study, we investigate the UAV remote-sensing route-planning problem in a huge remote-sensing region with predeployed PADs. We choose minimizing the total time it takes for a single UAV to execute a remote-sensing mission as the optimization objective and thus name this problem the PAD-based UAV remote-sensing time-minimization (PURSTM) problem. We first define and formulate the PURSTM problem and prove that this problem is NP-hard. Then, a route-planning scheme called the PAD-based remote-sensing (PBRS) scheme to schedule the UAV to travel to target points for a remote sensing mission is proposed. Simulation results show that our proposed scheme achieves significant performance.

The main contributions of this paper are as follows:

- By using predeployed PADs in the target region, we propose a novel solution to huge-region UAV remote sensing in which a single UAV can accomplish a huge-region remote-sensing mission in a single flight by automatically traveling to the PAD for energy replenishment. To the best of our knowledge, this is the very first study to introduce PAD technologies into the remote-sensing field.
- We propose the PURSTM problem with the goal of minimizing the total time spent on remote-sensing missions and examine how to plan the route of a single UAV to execute a remote-sensing mission over a huge region with predeployed PADs in a single flight. We formulate the problem and prove it is NP-hard.
- We propose a solution to the PURSTM problem called PBRS, which uses a more practical UAV model that accounts for the turning energy consumption and data storage of the UAV.
- We conduct several group simulations with real geographic scenarios to demonstrate the effectiveness of our proposed scheme.

The rest of the paper is organized as follows. Section 2 reviews the related work. Section 3 introduces the model and formulates our problem. Section 4 presents the proposed scheme. Section 5 conducts simulations for performance evaluation. Section 6 discusses the superiority of our proposed scheme. Section 7 concludes our work.

## 2. Related Work

In this section, we review existing research related to our work. First, we present research on the UAV route-planning problem for UAV remote sensing. Then, we discuss research on PAD.

### 2.1. Route-Planning Problem for UAV Remote Sensing

When planning a route for UAV remote sensing, it is necessary for the UAV to fly to each target point and back to the BS.

In the research on route planning for UAV remote sensing, the UAV has to fly to the target point, which is an important part of the route-planning algorithm for UAV remote sensing. Jang et al. [11] considered the route-planning problem for UAV remote sensing as a dynamically constrained traveling salesman problem (TSP) with neighborhoods. By incorporating the turning energy and switching energy into the UAV energy model, Huang et al. [12] were able to transform the UAV path-planning problem into a general target-visiting problem for minimizing the energy consumption of UAV flights based on waypoints. In [13], a modified mayfly algorithm based on an exponentially decreasing inertia weight strategy was proposed for UAV route planning in two-dimensional planar space. In [14], an improved butterfly optimization algorithm was proposed for three-dimensional space.

Some research projects have investigated deploying a UAV network consisting of multi-UAV operations as a method of addressing the limitations of single-UAV operations. Based on the vehicle-routing problem (VRP), Chen et al. [15] proposed an optimization algorithm for UAV route planning for remote-sensing observation, thereby minimizing the observation time and the number of UAVs dispatched. Xu et al. [16] used threat cost and fuel cost as criteria for evaluating routes. The algorithm also considered time and space constraints to improve mission efficiency and ensure the safety of the UAVs. Liu et al. [17] proposed a UAV swarm-scheduling method for the problem of multiple UAVs for remote-sensing observation, which improved the observation efficiency of the UAV swarm for emergency situations.

In addition, a few studies have begun to consider powering the UAV while it is in flight. In [18], the introduction of depots was proposed, thereby allowing UAVs to gain flight energy to visit more target points by traveling to the depot for recharging; however, the study deployed the depots by precomputing the route of the UAVs, which made it adaptable to only a small region with fewer target points. In [19], a deep reinforcement learning-based approach was proposed using an encoder–decoder-like policy network that uses two types of attention to learn the relationship between the monitoring target and the charging-station nodes. However, these studies deployed the charging stations randomly in the network, which cannot ensure that every target point in the region is accessible, and some of them might not be used, thereby resulting in wasted economic costs.

### 2.2. PAD Technology

With the expanding use of UAVs, there is a growing need for more efficient charging solutions. UAVs can autonomously recharge via either wired or wireless connections. Wired connections offer efficient energy use, but their reliability can be compromised due to the impact of environmental conditions on the system’s mechanical contacts. In contrast, wireless connections provide greater reliability but at the expense of efficiency. Wireless charging faces challenges in energy efficiency due to alignment issues between the receiver and transmitter circuit elements [20]. According to [21], the efficiency of wireless charging is typically 20% to 50% lower than that of wired charging. Nonetheless, the reliability offered by wireless charging is more suitable for the outdoor conditions of UAV remote-sensing operations. Moreover, as [21] notes, current PAD technology can achieve a wireless power transfer efficiency of 74.8%, with the potential to deliver a maximum charging power of up to 195*W* at an efficiency of 91% [22].

For economic cost savings and more efficient use of PADs in huge regions, Chen et al. [23] introduced PADs in wireless rechargeable sensor networks and proposed four heuristic algorithms for deploying PADs and an on-demand charging scheduling scheme for WRSNs, in which UAVs can charge uniformly distributed sensor nodes deployed in the network with PADs. However, Ref. [23] solely focused on the uniform distribution of central BSs and nodes, thus rendering it inadequate for large-scale or constrained node-deployment scenarios. Conversely, Ref. [24] investigated the problem of deploying a minimum number of PADs in UAV-based WRSNs and proposed a PAD deployment algorithm that can be applied to any BS location, any geographical distribution of sensor nodes, and any network region. In both [23,24], the deployment of PADs must satisfy two constraints simultaneously: the coverage constraint and the connectivity constraint. Among them, the coverage constraint requires that a node in the network be covered by at least one PAD, thereby ensuring that the UAV can reach the node via the PAD to replenish its energy. The connectivity constraint requires that the UAV must start from any PAD (including the BS) to reach another PAD in the network, which ensures that the UAV can start from the BS and successfully return to the BS after completing the mission.

## 3. Preliminaries

In this section, with the introduction of the region model and the energy-consumption model of the UAV, we formulate the PAD-based UAV remote-sensing time-minimization (PURSTM) problem and prove that it is NP-hard. Table 1 lists all the symbols used in this article.

### 3.1. Region Model

The original scene of the remote-sensing region is shown in Figure 2a: the region comprises one UAV, one BS, and *N* target points. The target points are randomly located in three-dimensional regions, thereby resulting in the UAV’s altitude being variable during flight. Let $O=\left\{o_{1},o_{2},\ldots ,o_{N}\right\}$ denote the set of target points. The location of each target point $o_{i}\in O\left(1\leq i\leq N\right)$ is known to both the BS and UAV. Without causing confusion, we also refer to $o_{i}$ as the coordinate of the corresponding target point.

The UAV needs to travel to the target point for remote-sensing missions by collecting images. The required number of images for target point $o_{i}$ is represented as $b_{i}$. Clearly, $b_{i}\in \left[b_{min},b_{max}\right]$, where $b_{min}$ and $b_{max}$ are the min number and the max number of images required for one target point, respectively. Let $B=\left\{b_{1},b_{2},\ldots ,b_{N}\right\}$ denote the set of the required number of images for all the target points.

The UAV is required to travel to these target points for remote sensing and then return to the BS or PAD for data offloading or recharging. Once the UAV has visited all of the target points to perform remote-sensing operations and returned to the BS, the remote-sensing mission is considered complete. To accomplish the remote-sensing mission in a cost-effective manner, we need to deploy as few PADs as possible in the target remote-sensing region. Therefore, we use the algorithm proposed in [24] to deploy *M* PADs across this region to accomplish coverage of all the target points, and the deployment result is shown in Figure 2b. For simplicity’s sake, we refer to the BS and PAD as charging stations because they can both supply power to the UAV and receive data from it. Let $P_{0}$ denote the BS, and let $P=\left\{P_{0},P_{1},P_{2},\ldots ,P_{M}\right\}$ denote the set of charging stations. We use $p_{i}\in P\left(0\leq i\leq M\right)$ for the location of the charging stations.

In our region model, we use the target-point locations in the remote-sensing region as the sensor-node positions in the algorithm in [24] to generate the corresponding PAD deployment. The coverage constraint and the connection constraint are used by the algorithm in [24] to determine where to place PADs. Both constraints become important assumptions in our case.

The coverage constraint requires that the UAV has enough energy to reach the target point for remote sensing and return to the nearest charging station to recharge, i.e., the distance between the target point and the nearest charging station can not greater than $d_{cover}$, which is formulated as follows:

(1) $$d\left(o_{i},p_{j}\right)\leq d_{cover},\forall o_{i}\in O,\exists p_{j}\in P$$

where $d\left(o_{i},p_{j}\right)$ is the Euclidean distance between $o_{i}$ and $p_{j}$.

The charging station $p_{j}$ is said to cover the target point $o_{i}$ if the constraint of Equation (1) is satisfied, and $p_{j}$ is the nearest charging station of $o_{i}$, which is denoted as follows:

(2) $$P\left(o_{i}\right)=p_{j},o_{i}\in C\left(p_{j}\right)$$

where $P\left(o_{i}\right)$ denotes the charging station covering $o_{i}$, and $C\left(p_{j}\right)$ denotes the set of target points covered by the charging station $p_{j}$. According to Equation (1), we have:

(3) $$O={⋃}_{i=1}^{N}C\left(p_{j}\right)$$

Meanwhile, the connectivity constraint requires that the distance between a charging station and its nearest charging station is not greater than $d_{max}$ so that the UAV has enough energy to travel from one charging station to the other, i.e., the UAV is able to travel from the BS and back to the BS to complete a remote-sensing mission. The connectivity constraint is expressed as follows:

(4) $$d\left(p_{i},p_{j}\right)\leq d_{max},\forall p_{i}\in P,\exists p_{j}\in P\setminus \left\{p_{i}\right\}$$

where $d\left(p_{i},p_{j}\right)$ denotes the Euclidean distance between $p_{j}$ and $p_{j}$. If $p_{i}$ and $p_{j}$ satisfy the constraint of Equation (4), then $p_{i}$ and $p_{j}$ are said to be neighbors, and, in the case that $p_{i}$ is a neighbor of $p_{j}$, for example, their relationship is expressed as follows:

(5) $$p_{i}\in N\left(p_{j}\right),p_{i}\in P,p_{j}\in P,i\neq j$$

where $N\left(p_{j}\right)$ denotes the set of charging stations that are close to the neighbors and excludes the others.

Based on the connectivity constraint, we can construct a weighted connectivity graph $G=\left(P,E\right)$ with the set of charging stations *P* as the set of vertexes and add the edge $e\left(p_{i},p_{j}\right)$ to the set of edges *E* with weight $w\left(p_{i},p_{j}\right)$ of the distance $d\left(p_{i},p_{j}\right)$ between $p_{i}$ and $p_{j}$ if they are neighbors. For the purpose of simplification, we assume that the energy of the charging stations is unlimited.

### 3.2. Energy Consumption Model

The UAV must fly to each target-point location within the region for its remote-sensing mission. We use a fixed constant ρ to represent the energy consumed by the UAV when it captures an image. δ denotes the size of each image taken. The energy consumed by the UAV for image acquisition at each target location is denoted by $e_{i}$, which is expressed as follows:

(6) $$e_{i}=b_{i}\rho$$

**Figure 3:**
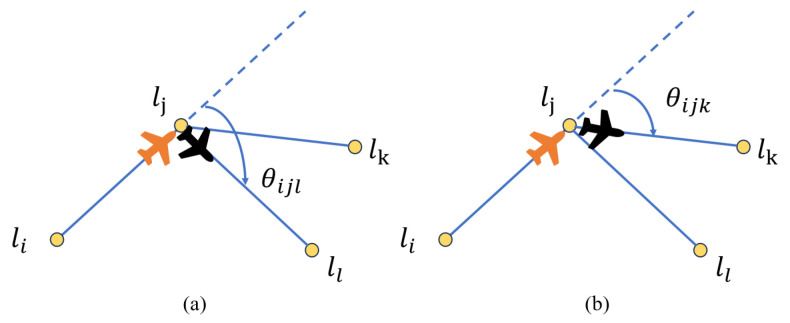
UAV turning schematic. (**a**) denotes the UAV traveling from point i via point j to point l and (**b**) denotes the UAV traveling from point i via point j to point k.

In most previous studies, the energy consumption of the UAV during flight was assumed to be directly proportional to the distance traveled, without consideration of the real-world circumstances. In actuality, the UAV must adjust its orientation during turns, which affects energy consumption and consequently influences the UAV’s chosen route. As depicted in Figure 3, when the UAV flies from point $l_{i}$ to point $l_{j}$, the distances to reach $l_{k}$ and $l_{l}$ are equal.

However, due to $\theta_{ijk}<\theta_{ijl}$, the energy consumption of routes with larger angles increases for the same distance traveled. Therefore, it is necessary to consider the energy consumption at corners in the flight route of a UAV. According to [12], the turning energy consumption $E_{turn}$ of a UAV flying to $l_{k}$, after reaching $l_{j}$ from $l_{i}$, can be expressed as follows:

(7) $$E_{turn}^{i,j,k}=\omega \theta_{i,j,k}+\beta$$

where ω and β are constant factors related to the turning energy consumption of the UAV. $\theta_{i,j,k}\left(0\leq \theta_{i,j,k}\leq \pi \right)$ signifies the turning angle of the UAV at position $l_{j}$, with its previous position being $l_{i}$ and the next one being $l_{k}$. This position could pertain to either the target point or the charging station.

Furthermore, according to [25], the turning time is calculated as follows:

(8) $$t_{turn}^{i,j,k}=\tau \theta_{i,j,k}$$

where τ is the angular velocity of the UAV.

The rotary-UAV energy model mentioned in [4] is used to calculate the traveling power $P_{mov}$ and hovering power $P_{hov}$ of the UAV. Based on this, we can determine the traveling energy consumption $E_{mov}$ and hovering energy consumption $E_{hov}$ of the UAV:

(9) $$E_{mov}=P_{mov}t_{mov}$$

(10) $$E_{hov}=P_{hov}t_{hov}$$

where $t_{mov},t_{hov}$ represent the traveling time and hovering time of the UAV in one remote-sensing mission, respectively.

As the UAV is required to hover at the target point for remote sensing, the number of images at each target point results in varying hovering times for the UAV. The hovering time of the UAV at target point $o_{i}$ is defined as follows:

(11) $$t_{h}^{i}=\eta b_{i}$$

where η is the time taken by the UAV to acquire an image; we obtain the total hovering time of the UAV:

(12) $$t_{hov}=\sum_{i=0}^{N}t_{h}^{i}+\frac{E_{UAV}}{P_{charge}}\times C_{return}$$

where $P_{charge}$ represents the power of the charging station to charge the UAV, and $C_{return}$ represents the number of times the UAV returns to the PAD.

Based on the moving speed $V_{U}$ and battery capacity $E_{UAV}$, the maximum travel distance $d_{max}$ is calculated as follows:

(13) $$d_{max}=\frac{E_{UAV}}{P_{mov}}\times V_{U}$$

Considering the additional energy consumption caused by the UAV for completing the remote sensing mission, the coverage distance $d_{cover}$ of the UAV can be calculated as follows:

(14) $$d_{cover}=\frac{1}{2}\times \frac{E_{UAV}-\rho b_{max}-2\times \left(\omega \pi +\beta \right)}{P_{mov}}\times V_{U}$$

### 3.3. Problem Definition

Considering the remote-sensing mission of the UAV, the UAV starts from the BS and ends back at the BS. Due to the energy limitation, the UAV travels to the charging stations for energy replenishment or data offloading due to energy shortage and data overflow, and it then travels to the remaining target points. Therefore, we can divide the whole route of UAV remote sensing into several subroutes. The subroutes start and end at charging stations. Since there may be many target points covered by a single charging station, making it impossible for the UAV to complete the remote sensing of the target points covered by that charging station at one time, there is a possibility that the end points and start points of the subroutes are the same charging station. Note that if the start and end charging stations of one subroute are different, they must be neighbors.

We denote the set of all subroutes as SP and the set of subroutes of a UAV from $p_{i}$ to $p_{j}$ as $SP_{i,j}$. Based on the above description, we can see that the following conditions must be satisfied for $SP_{i,j}$ to exist:

(15) $$p_{i}\in N\left(p_{j}\right)\cup \left\{p_{j}\right\}$$

where the *k*th subroute $sp_{{}_{i,j}}^{k}=\left\{o_{i,j}^{k,1},o_{i,j}^{k,2},\ldots ,o_{i,j}^{k,r}\right\}$ denotes a possible subroute from $p_{i}$ to $p_{j}$, and $o_{i,j}^{k,i}\left(1\leq i\leq r\right)$ denotes the *i*th target point in the subroute. Let $O_{i,j}^{k}$ be the set of target points contained in subroute $sp_{{}_{i,j}}^{k}$. According to the UAV model mentioned above, the time of the UAV flying along a subroute is expressed as follows:

(16) $$Time_{i,j}^{k}=t_{mov}+t_{hov}+t_{turn}$$

where $t_{turn}$ denotes the time spent by the UAV to turn while traveling in $sp_{{}_{i,j}}^{k}$.

Meanwhile, the energy consumption of the UAV in $sp_{{}_{i,j}}^{k}$ can be calculated as follows:

(17) $$Cost_{i,j}^{k}=P_{mov}t_{mov}+P_{hov}t_{hov}+turn_{i,j}^{k}+\rho \times image_{i,j}^{k}$$

where $turn_{i,j}^{k},image_{i,j}^{k}$ denote the turning energy consumption and the total number of images captured by the UAV traveling along $sp_{{}_{i,j}}^{k}$, respectively.

The decision variable $x_{i,j}^{k}$ is defined below:

(18) $$x_{i,j}^{k}=\left\{\begin{array}{cc} & 1\;\mathrm{if}\;\mathrm{the}\;\mathrm{UAV}\;\mathrm{flies}\;\mathrm{along}\;\mathrm{the}\;\mathrm{subroute}\;sp_{i,j}^{k}\\ \; & 0\;\;otherwise \end{array}\right.$$

We consider the case where there is a direct flight from one charging station to another during the UAV flight, i.e., there exists $sp_{{}_{i,j}}^{k}=⌀$ such that $O_{i,j}^{k}=⌀$. To simplify the description, we define the following computation:

(19) $$x_{i,j}^{k}*O_{i,j}^{k}=\left\{\begin{array}{cc} \; & O_{i,j}^{k}\;\;\mathrm{if}\;x_{i,j}^{k}=1\\ \; & ⌀\;\;\;\;\;otherwise \end{array}\right.$$

Therefore, the total time required by the UAV to complete a remote-sensing mission can be calculated using the following equation:

(20) $$T=\sum_{SP_{i,j}\in SP}^{}\sum_{sp_{{}_{i,j}}^{k}\in SP_{i,j}}x_{{}_{i,j}}^{k}Time_{i,j}^{k}$$

Our goal is to plan a route such that *T* is minimized. Based on the above, our problem is defined as follows:

**Definition** **1.** (The Proplem of PAD-based UAV Remote Sensing with Minimized Energy Consumption, PURSTM). *Given a network region *Ω*, a set of target points $O=\left\{o_{1},o_{2},\ldots ,o_{N}\right\}$, a set of charging stations $P=\left\{p_{0},p_{1},\ldots ,p_{M}\right\}$, the objective is to find the UAV flight route that achieves the remote-sensing mission with minimum energy consumption. The problem can be formulated as follows:*

(21) objective:minT

(22) $$s.t.Cost_{i,j}^{k}\leq E_{UAV}$$

(23) $$image_{i,j}^{k}\delta \leq B_{UAV}$$

(24) $$\sum_{SP_{i,j}\in SP}^{}\sum_{sp_{{}_{i,j}}^{k}\in SP_{i,j}}\left|x_{{}_{i,j}}^{k}*O_{i,j}^{k}\right|=N$$

(25) $${⋃}_{SP_{i,j}\in SP}{⋃}_{sp_{{}_{i,j}}^{k}\in SP_{i,j}}x_{{}_{i,j}}^{k}*O_{i,j}^{k}=O$$

(26) $$\sum_{SP_{0,j}\in SP}^{}\sum_{sp_{{}_{0,j}}^{k}\in SP_{0,j}}x_{{}_{0,j}}^{k}=\sum_{SP_{i,0}\in SP}^{}\sum_{sp_{{}_{i,0}}^{k}\in SP_{i,0}}x_{{}_{i,0}}^{k}\geq 1$$

Constraints (22) and (23) ensure that all possible subroutes cannot exceed the energy and storage limit of the UAV, and Constraints (24) and (25) ensure that the UAV travels to all the target points to capture the images and travels to them only once in completing the remote-sensing mission. Constraint (26) demonstrates that the UAV departs from the BS and eventually returns to the BS.

**Theorem** **1.** *The PURSTM problem is NP-hard.*

**Proof** **of Theorem 1.** Assuming that the energy and storage of the UAV are infinite, the UAV will not need to return to the charging station during the remote-sensing mission. In this case, the PURSTM problem becomes a problem of finding a route that can traverse all the target points to minimize *T*, starting and ending at the BS. According to Equation (20), the number of images captured at each target point is constant, so *T* is related to $t_{mov}$ and $t_{turn}$. According to [12], we can construct a directed weighted graph by discretizing the turning angles, thereby providing a finite set of angle options using the weights of the edges to represent the turning time. The problem is transformed into a generalized traveling salesman problem (GTSP) problem, which was shown to be NP-hard in [12]. Thus, the PURSTM problem is a GTSP problem without considering energy and memory, and since the GTSP problem is NP-hard, the PURSTM problem is NP-hard.   □

## 4. Proposed Scheme

As described in Section 3.3, the PAD must satisfy the connectivity constraint and the coverage constraint in the remote-sensing region, where each target point is covered by a charging station, and the charging stations are connected. For any target point $o_{}^{i}$, the UAV can travel from charging station $P\left(o_{}^{i}\right)$ to $o_{}^{i}$ for the remote-sensing mission. Based on the above considerations, we propose the PAD-based remote-sensing (PBRS) scheme to solve the PURSTMP problem.

**Figure 4:**
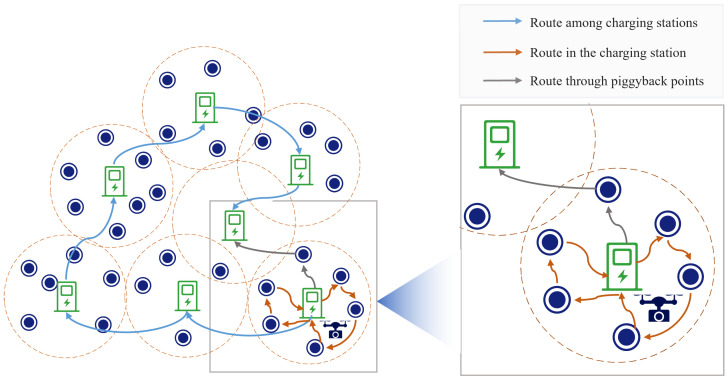
Three flight routes for UAVs in the PBRS scheme.

The PBRS scheme constructs the route of the UAV based on the locations of the PADs, and the main idea is as follows: First, a loop is constructed that starts from the BS and returns to the BS after traveling to all the PADs. Then, the UAV travels along this loop, and when the UAV reaches each a PAD, it travels to all the target points covered by that PAD for remote sensing. If the UAV has enough energy to travel to the nearby target points covered by the nearest charging station that have not yet completed the remote-sensing mission, it will travel to the remaining target points covered by that charging station and then fly directly to the next charging station of the loop. In the PBRS scheme, these target points are called piggyback points. Three flight routes for UAVs in the PBRS scheme are shown in Figure 4.

In Section 3.1, a connected graph $G=\left(P,E\right)$ was created using charging stations, but there is no guarantee that it is a Hamiltonian graph. To obtain the shortest loop, we generated a weighted complete graph $G^{\prime }$ and obtained our loop based on the result of the TSP algorithm.

**Figure 5:**
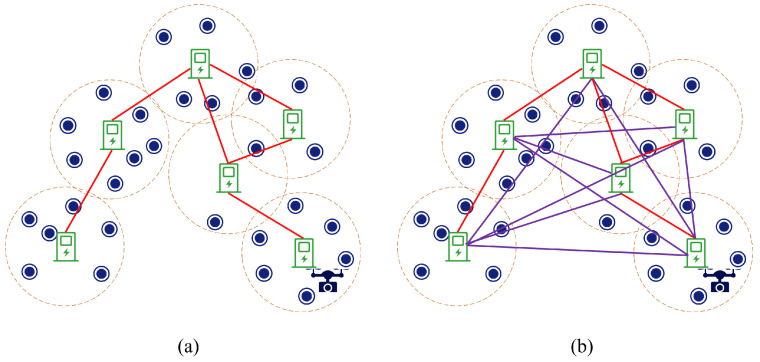
Complete graph (**b**) constructed based on (**a**); the purple lines are the newly added edges for $G^{\prime }$.

A weighted complete graph $G^{\prime }=\left(P,E^{\prime }\right)$ based on $G=\left(P,E\right)$ was constructed. As shown in Figure 5, we first added the set of edges *E* to $E^{\prime }$. Then, for the charging station $p_{i}$, we checked the edge $e\left(p_{i},p_{j}\right)\in E,i\neq j$, and if it was not satisfied, we added the edge $e\left(p_{i},p_{j}\right)$ to $E^{\prime }$ and set the weight $w\left(p_{i},p_{j}\right)$ as the length of $shortestpath\left(p_{i},p_{j}\right)$ computed in *G*. After conducting the above operations for each charging station in *P*, we obtained the weighted complete graph $G^{\prime }$.

We ran the Lin–Kernighan–Helsgaun(LKH) algorithm based on $G^{\prime }$ to find the shortest Hamiltonian loop. Then, each edge $e\left(p_{i},Succ\left(p_{i}\right)\right),i=0,\ldots ,M$ was required to be checked in this loop, where $Succ\left(p_{i}\right)$ became the successor of $p_{i}$ in the loop. If $e\left(p_{i},Succ\left(p_{i}\right)\right)\notin E$, we replaced the edge in the loop with $shortestpath\left(p_{i},p_{j}\right)$. Finally, a valid loop comprising all the charging stations could be obtained.

After achieving a valid loop, we established the sequence for the UAV visiting the charging stations. The following step involved identifying piggyback points between adjacent charging stations in a specific sequence. To include more target points as potential piggyback points, we initially created an ellipse with $p_{i}$ and $p_{j}$ as focal points. The long axis length is determined as follows:

(27) $$a_{ellipse}=\frac{E_{rem}-\omega \pi -\beta -P_{hov}b_{max}\mu -b_{max}\rho }{P_{mov}}\times V_{U}$$

The ellipsoid was generated by rotating the constructed ellipse around the axes defined by the two focal points; since the UAV departs from $p_{i}$ with full energy, we have $E_{rem}=E_{UAV}$. According to the properties of the ellipsoid, it can travel to any target points within this ellipsoid for remote sensing before traveling to $p_{j}$. If multiple target points fall within the ellipsoid, the UAV chooses the target point $o_{p}$ that is closest to the charging station $p_{i}$ to be the piggybacking point. Then, using $o_{p}$ and $p_{j}$ as focal points, we proceeded to construct the ellipsoid through the aforementioned method in order to locate the subsequent piggyback point. Whether the construction of the ellipsoid is valid or not depends on the success of the ellipse’s construction. Therefore, it is necessary to check that the length of the long axis is greater than the new focal length. If so, the above operation will be repeated until no ellipsoid can be constructed or until there are no more target points within the current ellipsoid. Finally, all piggyback points for a remote sensing mission were determined.

After determining the piggyback points, we considered the charging process of the UAV in a subregion of individual charging stations. Upon reaching the charging station $p_{i}$, the UAV will travel to the non-piggyback point in $p_{i}$ for remote sensing. According to [26], the route-planning problem of UAV in this subregion can be considered as a VRP problem. We solved this problem using a genetic algorithm to obtain the final route of the UAV. The details of the PBRS scheme are depicted in Algorithm 1.
**Algorithm 1:** PAD-based remote-sensing scheme
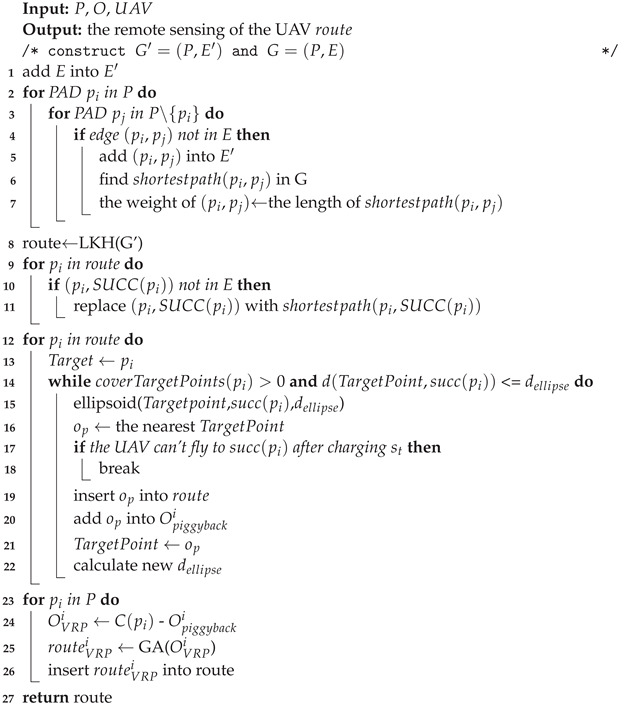


## 5. Experimental Simulation

In this section, we evaluate the performance of our proposed scheme. To better showcase the performance of our algorithm, we introduce a very basic route-planning scheme, known as target point-based remote sensing (TBRS), for comparison, as no existing studies align with our mission scenario. The TBRS scheme plans the flight route of the UAV based on the location of the target points. It constructs a Hamiltonian loop based on the BS and the locations of all the target points while ignoring the energy and storage limits of the UAV; it then checks the energy and storage of the UAV before each target point and inserts the nearest PAD if the energy and storage constraints of the UAV are not satisfied to obtain a final route.

To assess the performance of the proposed scheme, we conducted four sets of simulation experiments by varying the number of target points, the size of the region, the energy capacity of the UAV, and the storage capacity of the UAV.

The reasons for selecting these four variables are as follows:

(1) The number of target points significantly impacts the scale of UAV missions for remote sensing, thus resembling a node-density consideration. By adjusting the number of target points, we evaluate the scheme’s performance across varying mission scales.
(2) Simultaneously, the size of the region directly affects mission range and complexity, thereby posing a fundamental challenge akin to node density. Altering the size of the region allows us to gauge the scheme’s adaptability to diverse environmental conditions.
(3) The energy capacity of the UAV determines its endurance for flight. By varying the energy capacity, we can evaluate the performance of the scheme under different endurance requirements and assess its feasibility for prolonged remote-sensing missions [27]. Meanwhile, changes in the energy capacity of the UAV can influence the deployment of PADs [24], thus altering the network topology. Studying the impact of energy variations on the performance of UAVs completing remote-sensing missions in this scenario is crucial for understanding the scheme’s robustness.
(4) Due to the constraints in our mathematical model, the UAV will travel to the charging station for data offloading when faced with insufficient storage space. Nevertheless, in practical scenarios, we could address storage issues by replacing the storage card of the camera on the UAV. For the sake of the completeness of our model, we incorporate this factor into consideration.

We evaluated the performance of the scheme through three metrics: the duration of remote sensing, the frequency of visits to the charging station, and the data-storage rate of the UAV. Our objective was to optimize the remote-sensing duration to achieve higher efficiency. A shorter remote-sensing time signifies improved performance of the scheme. The trips to the charging station correlate with the frequency of the UAV for charging or data offloading. Therefore, a reduced frequency results in a shorter remote-sensing time, thus demonstrating the enhanced performance of the algorithm. The data-storage rate, quantified as the ratio of stored data to the UAV’s storage capacity during charging-station visits, holds a pivotal role. A heightened data-storage rate reflects enhanced efficiency in executing remote-sensing missions, thereby demonstrating the scheme’s performance in planning more efficient routes.

### 5.1. Setup of the Simulation Environment

**Figure 6:**
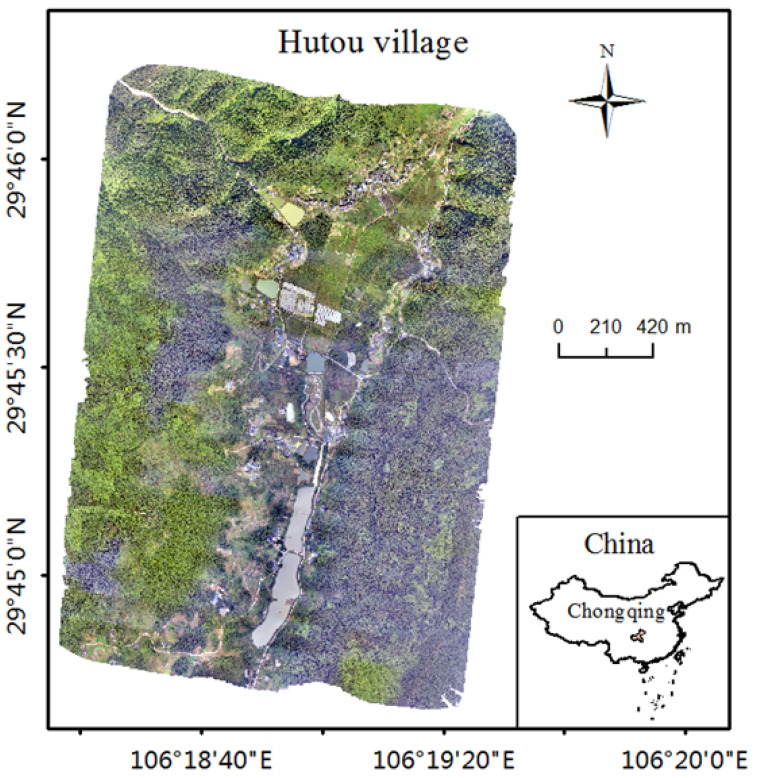
Digital orthophoto model of Hutou Village in Beibei District, Chongqing, China.

To demonstrate how our proposed scheme could be put into practice, we selected the geographic data of Hutou Village in Beibei District, Chongqing City, China, which is located at 106.33 degrees east longitude and 29.76 degrees north latitude with a width of 2034.6 m and a height of 2861.2 m, as the simulation environment, i.e., the remote-sensing region model for the simulation experiments, and the digital orthophoto model of Hutou Village is shown in Figure 6.

### 5.2. Impact of the Number of Target Points

In each simulation, target points were randomly distributed across the region, with the exception of experiments exploring variations in region sizes. Specifically, in simulations with different region sizes, the BS was centrally positioned within the rectangular region. The region’s size was adjusted by manipulating the side length of the rectangular region. The center of the region consistently served as the designated position for the BS across all experimental setups. The locations of the PADs were determined using the deployment algorithm mentioned in [24]. The parameters utilized for these simulation experiments are detailed in Table 2.

**Figure 7:**
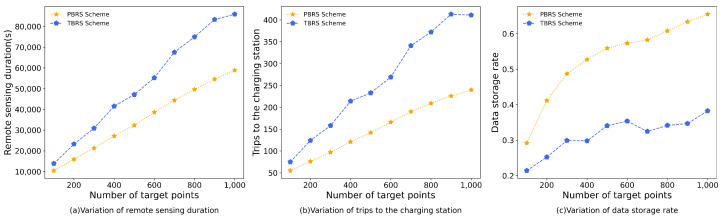
The simulation results of varying the number of target points.

Figure 7 depicts the simulation results for increasing the number of target points from 100 to 1000. According to [24], the variation in the number of target points does not have a significant effect on the number of PADs deployed, since the number of PADs is mainly limited by the coverage of the UAV and the size of the region. In this simulation, the remote-sensing duration for the PBRS scheme was reduced by 48.26% compared to the TBRS scheme. Additionally, the number of trips to the charging station decreased by 71.48%, and there was a 69% increase in the data-storage rate.

According to Figure 7, it is evident that the remote-sensing duration, as well as the trips to the charging station, tends to increase as the number of target points increases. This is due to the fact that the increase in the number of target points leads to an increase in the amount of data for remote sensing, which prolongs the remote-sensing duration and increases the trips to the charging station. With both metrics evaluated, the PBRS scheme outperformed the TBRS scheme. This is because in the TBRS scheme, the route of the UAV is adapted from a Hamiltonian loop based on the location of target points. The UAV may shuttle between different charging stations due to the case that multiple adjacent target points on the initial route are not covered by the same charging station, thus significantly prolonging the remote-sensing duration and causing the UAV to travel to the charging stations repeatedly for energy replenishment. In the PBRS scheme, the UAV travels to all the target points covered in a charging station before traveling to the next, which saves a lot of time traveling between charging stations, thus resulting in fewer trips to the charging stations. While the PBRS scheme has a shorter remote-sensing time and fewer trips to charging stations compared to the TBRS scheme, the UAV also has a higher data-storage rate. This difference is attributed to the fact that in the TBRS scheme, the UAV constructs the route based on target points; there are cases where the UAV only travels to a node for remote sensing and then travels to a charging station that is more distant from it or even travels to a charging station without traveling to the nearby nodes directly, thereby resulting in a lower data-storage rate for the UAV than for the PBRS scheme.

### 5.3. Impact of the Size of Region

**Figure 8:**
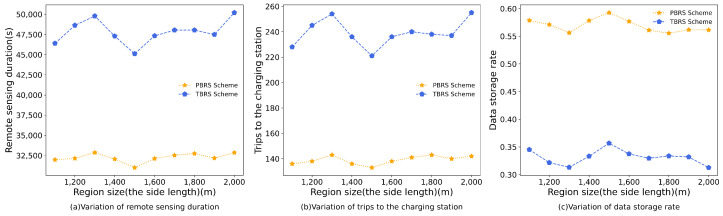
The simulation results of varying the size of region.

Figure 8 shows the simulation results for varying the remote-sensing region sizes, with side lengths growing from 1100 m to 2000 m; this is because the terrain data we use is not a regular rectangle but an irregular polygon with a maximum width of about 2000 m. In this simulation, the remote-sensing duration for the PBRS scheme was reduced by 48.33% compared to the TBRS scheme. Additionally, the number of trips to the charging stations decreased by 71.94%, and there was a 71.75% increase in the data-storage rate.

Unlike the variation in the number of target points, PAD deployment is influenced by the size of the region, as it is evident that the need for more PADs increases with larger remote-sensing regions. Based on the data from Figure 8a,b, we identified rising trends in both the remote-sensing duration and the trips to the charging stations as the remote-sensing region expanded. However, some fluctuations occurred, which can be attributed to changes in the number of deployed PADs resulting from the variation in the size of the remote-sensing region. When the region is limited, there are only a few deployed PADs. In such situations, increasing the size of the remote-sensing region results in an increased number of deployed PADs. This allows for closer proximity of the PADs to each target point, thereby enabling UAVs to travel shorter distances to return to nearby charging stations after remote sensing and reducing the traveling time. The growth in the remote-sensing duration can be attributed to the region’s expansion, thereby resulting in a decrease in target-point density. As a result, the average distance for the UAV travel to each target point increases, thereby leading to longer travel. This, in turn, results in an increased number of trips to the charging stations, as illustrated in Figure 8b. Since the number of target points in the region and the amount of sensed data remained constant, an increase in the trips to the charging stations led to a decline in the average amount of data unloaded per trip to the charging stations, as illustrated in Figure 8c.

According to the findings depicted in Figure 8, it can be concluded that the PBRS algorithm outperformed the TBRS scheme.

### 5.4. Impact of the Energy Capacity of the UAV

**Figure 9:**
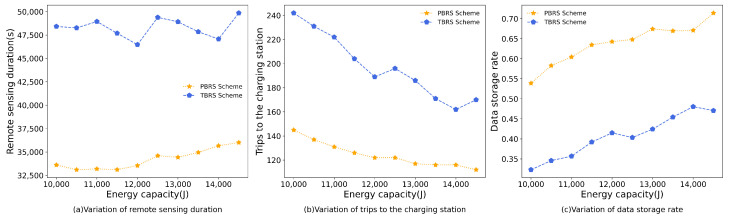
The simulation results of the energy capacity of the UAV.

In this simulation, the energy capacity of the UAV was increased from 10,000 J to 14,500 J. The remote-sensing duration for the PBRS schem was reduced by 41.09% compared to the TBRS scheme. Additionally, the number of trips to the charging stations decreased by 58.60%, and there was a 56.97% increase in the data-storage rate. The simulation results of two schemes are shown in Figure 9.

The energy-consumption trends for both the TBRS scheme and the PBRS scheme in Figure 9a exhibit fluctuations in an upward direction. The reason for this observation is that [24] takes into account the cost required to deploy PADs and seeks to use the minimum number of PADs in the region to enable the UAV to accomplish the mission. In [24], the energy capacity of the UAV determines the $d_{cover}$ and $d_{max}$. The higher the energy of the UAV, the larger $d_{cover}$ and $d_{max}$, which will change the number of PADs deployed and their locations. The increased energy of the UAV leads to a decrease in the number of PADs deployed, which means that each PAD covers more target points. And it also leads to an increase in the distance between charging stations and a greater distance between the target points and the nearest PADs, which increases the traveling time of the UAV. Meanwhile, Figure 9b illustrates a reduction in the trips to the charging stations for both algorithms as the battery capacity increased. This is due to the UAV’s increased energy capacity, which allows it to travel to more target points for remote sensing in a single flight without exceeding its storage capacity. As a result, the trips to the charging stations decreased while the data-storage rate of the UAV increased, as shown in Figure 9c.

Based on the results of Figure 9, it can be concluded that the PBRS algorithm outperformed the TBRS scheme.

### 5.5. Impact of the Storage Capacity of the UAV

**Figure 10:**
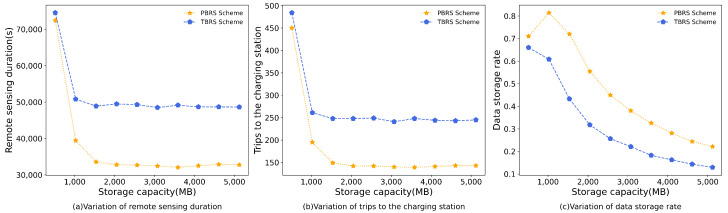
The simulation results of the storage capacity of the UAV.

In the experiment of UAV storage capacity, we increased the storage capacity from 512 MB to 5 GB. In this simulation, the remote-sensing duration for the PBRS scheme was reduced by 38.29% compared to the TBRS scheme. Additionally, the number of trips to the charging stations decreased by 51.96%, and there was a 50.97% increase in the data-storage rate. Figure 10 shows the simulation results of the two schemes.

It can be seen that the simulation results of the two algorithms were very similar when the storage capacity is 512 MB. This is due to the UAV’s limited storage capacity, which forces it to travel to a single target point for remote sensing. This resulted in the UAV wasting a lot of time on the route, regardless of whether there was still energy left, thus resulting in similar performance outcomes of the two schemes. As the UAV storage capacity increased, both the remote-sensing duration and the trips to the charging stations showed a clear downward trend at the outset before plateauing thereafter, as indicated by Figure 10a,b. This trend can be attributed to the fact that, as storage capacity increases, energy capacity becomes the main constraint on UAV flight. Figure 10c illustrates that the data-storage rate of the UAV decreased as its storage capacity increased. Our previous analysis showed that energy limitations constrain UAV flights, and the amount of data collected in a single flight to the target point does not change significantly, thereby resulting in a decrease in the data-storage rate of the UAV.

The results from the simulation depicted in Figure 10 are consistent with those obtained in the previous three experiments and demonstrate the superior performance of the PBRS scheme over the TBRS scheme in all three aspects.

## 6. Discussion

**Figure 11:**
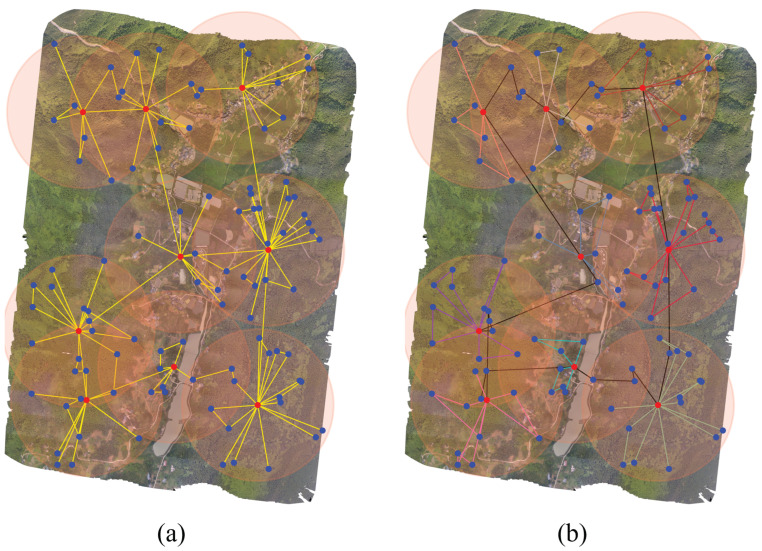
The route results. (**a**) shows the route of the TBRS scheme, and (**b**) shows the route of the PBRS scheme.

Combining the results of the four simulation experiments, we found that our proposed PBRS scheme consistently outperformed the TBRS scheme. We take the simulation experiment in Section 5.2 with the number of target points of 100 as an example. The routes of the TBRS and PBRS schemes are shown in Figure 11.

Figure 11a illustrates that the TBRS scheme contained numerous target points that the UAV traveled to and then directly to the adjacent charging station without traveling to other nodes, which increased the flight route, thus increasing the traveling time of the UAV and also causing an increase in the trips to the charging station. Conversely, in Figure 11b, black lines represent the UAV’s path from one charging station to another, either directly or via piggyback points. The lines in various colors signify the UAV’s remote-sensing routes within different charging stations. It is evident that the UAV visited multiple target points for remote sensing on each subroute, which resulted in shorter flight routes, thereby reducing both the sensing time and the number of trips to charging stations. This efficiency in the PBRS method is attributed to two factors. First, the use of piggyback points in the PBRS scheme optimizes the UAV’s travel route. Second, a more realistic energy consumption model for the UAV is employed in the PBRS scheme, thereby focusing on optimizing its turning energy consumption. The simulation results demonstrate that the PBRS scheme we propose can be employed in extensive UAV remote-sensing missions and exhibits excellent performance.

Furthermore, considering the three existing methods, let us examine the first one: assuming the terrain is obstacle-free, we hypothesize that the BS’s movement speed is 5 m/s and its redeployment time is 5 min. It will result in an additional time of about 64.45% of the remote-sensing time, which is unacceptable for remote-sensing missions. In the second method, according to Figure 11, nine extra UAVs and nine additional BSs are required, thereby significantly increasing economic costs. Additionally, damage to any one UAV could severely compromise the remote-sensing outcomes. According to [28] through mathematical modeling, the comparative study of communication architectures, routing protocols, and the decision-making roadmap in multi-UAV systems require resolution. The technical implementation faces a significant challenge. If the third method is used, in order to cover all the target points in the region, the battery capacity of the UAV needs to be increased, and according to [29], assuming the use of Ni-Cd batteries, the weight of the UAV’s batteries will increase by approximately three times, and the volume of the batteries will increase by approximately 51.84 cm^3^, which will result in a consequent increase in the volume of the UAV; in order to carry a larger volume of the batteries, the UAV’s own weight will increase, which drastically increases the energy consumption rate of the UAV. At the same time, this adds an additional element of unexpectedness to the flight control of the UAV.

## 7. Conclusions

Our work introduced a new idea using PADs in the field of huge-region UAV remote sensing. This innovative approach may change the way in which huge-region remote-sensing missions are executed. We enabled a single UAV to accomplish a significant remote-sensing operation in one continuous flight by using predeployed PADs inside the target region such that the UAV can fly autonomously to the PADs for energy replenishment without human intervention. The incorporation of PADs not only improved efficiency, but also expanded the possibilities for huge-region UAV remote sensing. The PURSTM problem was the result of our investigation into the nuances of this novel approach. In order to carry out remote-sensing missions across large territories using predeployed PADs, the optimized route must be planned while minimizing the UAV’s total energy. We proposed the PBRS scheme, which accounts for real-world constraints such as the UAV’s limited capacity for storing and using data, to provide an efficient solution to the PURSTM problem. We used real-world geographical conditions in a series of group simulations to prove the effectiveness of the proposed scheme. These simulations were essential proof that our method is applicable and useful in practice.

In addition, the PAD deployment approach has a major effect on the performance of UAV route-planning algorithms for huge-region remote sensing. We directly used the PAD deployment scheme originally used for WRSNs in our work, and some assumptions of this scheme, such as the assumption that a PAD can be deployed at any location in the target region, may not be valid in some remote-sensing domains due to terrain constraints, e.g., in forest remote sensing, the UAV may not be able to hover and recharge over the PAD due to ground-vegetation obstruction. As a result, our future research will focus on PAD deployment approaches that are tailored to the topographical features of various target regions for remote-sensing applications. Moreover, the concurrent involvement of multiple UAVs in simultaneous remote-sensing missions stands poised to substantially enhance mission efficiency. This improvement, however, necessitates a thorough quantification of the supplementary power requirements linked to the inclusion of each UAV in the charging infrastructure when utilizing the same docking station. This critical analysis is imperative for understanding and optimizing the collective power demands inherent in the simultaneous operation of multiple UAVs, thereby contributing to the ongoing efforts to enhance mission effectiveness.

## Figures and Tables

**Table 1:** List of notations.

Notation	Description
$E_{UAV}$	Battery capacity of the UAV
$B_{UAV}$	Buffer capacity of the UAV
$V_{U}$	Flight speed of the UAV
*O*	The set of target points
*P*	The set of charging stations
*B*	The set of the number of images required for the target points
$E_{mov}$	Energy consumption during the movement of the UAV
$E_{hov}$	Energy consumption of the UAV hovering
$E_{turn}$	Energy consumption of the UAV turning
*E*	The sum of $E_{mov}$ and $E_{hov}$ and $E_{turn}$
$P_{hov}$	The hovering power of the UAV
$P_{mov}$	The motion power of the UAV
$P_{charge}$	The power of charging station to charge the UAV
$b_{max}$	The max number of images required for one target point
$b_{min}$	The min number of images required for one target point
$C_{return}$	The number of times the UAV returns to the PAD
η	Time cost of the UAV to collect an image
δ	Data size of an image
β	Constant of energy consumption of the UAV turning
ω	Coefficient of energy consumption of the UAV turning
ρ	Energy consumption of the UAV to acquire an image
τ	The angular velocity of the UAV
$t_{mov}$	The total flight time of the UAV
$t_{hov}$	The total hovering time of the UAV
$d_{max}$	The maximum flight distance of the UAV
$d_{cover}$	The charging coverage of the UAV
$t_{i}$	The position of the target point i or the PAD i
$\theta_{ijk}$	The angle of turn required for the UAV to travel from point i to j to k

**Table 2:** Default parameters.

Parameter	Value
Number of target points	500
BS location	central
$E_{UAV}$	10,000 J
$B_{UAV}$	2 GB
$V_{U}$	20 m/s
$\left[b_{min},b_{max}\right]$	[10,20]
η	0.05 s
δ	2.57 MB
β	104.65 J
ω	5.3316
ρ	50 J

## Data Availability

Data are contained within the article.

## Abbreviations

The following abbreviations are used in this manuscript:

UAV	Unmanned aerial vehicle
WRSN	Wireless rechargeable sensor network
TSP	Traveling salesman problem
VRP	Vehicle-routing problem
BS	Base station
LKH	Lin–Kernighan–Helsgaun
PBRS	PAD-based remote sensing
TBRS	Target-based remote sensing
PURSTM	PAD-based UAV remote sensing time minimization
GTSP	Generalized traveling salesman problem

## References

[1] He J., Lin J., Zhang X., Liao X. (2023). Accurate estimation of surface water volume in tufa lake group using UAV-captured imagery and ANNs. Measurement.

[2] Zhang H., Li Y., Yang Y., Feng Y., Li Y., Deng C., Yuan D. (2023). UAV Tracking Based on Correlation Filters With Dynamic Aberrance-Repressed Temporal Regularizations. IEEE J. Sel. Top. Appl. Earth Obs. Remote Sens..

[3] Zhu F., Li H., Li J., Zhu B., Lei S. (2023). Unmanned aerial vehicle remote sensing image registration based on an improved oriented FAST and rotated BRIEF-random sample consensus algorithm. Eng. Appl. Artif. Intell..

[4] Zeng Y., Xu J., Zhang R. (2019). Energy Minimization for Wireless Communication With Rotary-Wing UAV. IEEE Trans. Wirel. Commun..

[5] Jones M.R., Djahel S., Welsh K. (2023). Path-Planning for Unmanned Aerial Vehicles with Environment Complexity Considerations: A Survey. ACM Comput. Surv..

[6] Wang M., Lin J. (2020). Retrieving individual tree heights from a point cloud generated with optical imagery from an unmanned aerial vehicle (UAV). Can. J. For. Res..

[7] Javaid S., Saeed N., Qadir Z., Fahim H., He B., Song H., Bilal M. (2023). Communication and Control in Collaborative UAVs: Recent Advances and Future Trends. IEEE Trans. Intell. Transp. Syst..

[8] Simic M., Bil C., Vojisavljevic V. (2015). Investigation in Wireless Power Transmission for UAV Charging. Procedia Comput. Sci..

[9] Choi C.H., Jang H.J., Lim S.G., Lim H.C., Cho S.H., Gaponov I. (2016). Automatic wireless drone charging station creating essential environment for continuous drone operation. Proceedings of the 2016 International Conference on Control, Automation and Information Sciences (ICCAIS).

[10] Costea I.M., Pleşca V. (2018). Automatic battery charging system for electric powered drones. Proceedings of the 2018 IEEE 24th International Symposium for Design and Technology in Electronic Packaging (SIITME).

[11] Jang D., Chae H., Choi H. Optimal Control-Based UAV Path Planning with Dynamically-Constrained TSP with Neighborhoods. Proceedings of the 2017 17th International Conference on Control, Automation and Systems (ICCAS).

[12] Huang J., Shan F., Xiong R., Shao Y., Luo J. (2021). Energy-Efficient UAV Flight Planning for a General PoI-Visiting Problem with a Practical Energy Model. Proceedings of the 30th International Conference on Computer Communications and Networks, ICCCN 2021.

[13] Wang X., Pan J.S., Yang Q., Kong L., Snášel V., Chu S.C. (2022). Modified mayfly algorithm for UAV path planning. Drones.

[14] Luo Y., Lu J., Zhang Y., Zheng K., Qin Q., He L., Liu Y. (2022). Near-Ground Delivery Drones Path Planning Design Based on BOA-TSAR Algorithm. Drones.

[15] Chen X., Li Q., Li R., Cai X., Wei J., Zhao H. (2023). UAV Network Path Planning and Optimization Using a Vehicle Routing Model. Remote Sens..

[16] Xu C., Xu M., Yin C. (2020). Optimized multi-UAV cooperative path planning under the complex confrontation environment. Comput. Commun..

[17] Liu J., Liao X., Ye H., Yue H., Wang Y., Tan X., Wang D. (2022). UAV Swarm Scheduling Method for Remote Sensing Observations during Emergency Scenarios. Remote Sens..

[18] Sundar K., Rathinam S. (2014). Algorithms for Routing an Unmanned Aerial Vehicle in the Presence of Refueling Depots. IEEE Trans. Autom. Sci. Eng..

[19] Fan M., Wu Y., Liao T., Cao Z., Guo H., Sartoretti G., Wu G. (2023). Deep Reinforcement Learning for UAV Routing in the Presence of Multiple Charging Stations. IEEE Trans. Veh. Technol..

[20] Campi T., Cruciani S., Feliziani M., Maradei F. High efficiency and lightweight wireless charging system for drone batteries. Proceedings of the 2017 AEIT International Annual Conference.

[21] Ucgun H., Yuzgec U., Bayilmis C. (2021). A review on applications of rotary-wing unmanned aerial vehicle charging stations. Int. J. Adv. Robot. Syst..

[22] Song Y., Sun X., Wang H., Dong W., Ji Y. Design of Charging Coil for Unmanned Aerial Vehicle-Enabled Wireless Power Transfer. Proceedings of the 2018 8th International Conference on Power and Energy Systems (ICPES).

[23] Chen J., Yu C.W., Ouyang W. (2020). Efficient Wireless Charging Pad Deployment in Wireless Rechargeable Sensor Networks. IEEE Access.

[24] Chen Y., Gu Y., Li P., Lin F. (2021). Minimizing the number of wireless charging PAD for unmanned aerial vehicle-based wireless rechargeable sensor networks. Int. J. Distrib. Sens. Netw..

[25] Chen H., Wang X., Shen L., Li Z., Liu Z., Yu Y. (2021). Formation Reconfiguration for Fixed-Wing UAVs. J. Intell. Robot. Syst..

[26] Dai R., Fotedar S., Radmanesh M., Kumar M. (2018). Quality-aware UAV coverage and path planning in geometrically complex environments. Ad Hoc Netw..

[27] Murray C.C., Chu A.G. (2015). The flying sidekick traveling salesman problem: Optimization of drone-assisted parcel delivery. Transp. Res. Part C Emerg. Technol..

[28] Adoni W.Y.H., Lorenz S., Fareedh J.S., Gloaguen R., Bussmann M. (2023). Investigation of Autonomous Multi-UAV Systems for Target Detection in Distributed Environment: Current Developments and Open Challenges. Drones.

[29] Boukoberine M.N., Zhou Z., Benbouzid M. (2019). A critical review on unmanned aerial vehicles power supply and energy management: Solutions, strategies, and prospects. Appl. Energy.

